# Risk factors for pneumothorax in advanced and/or metastatic soft tissue sarcoma patients during pazopanib treatment: a single-institute analysis

**DOI:** 10.1186/s12885-016-2786-z

**Published:** 2016-09-23

**Authors:** Kenji Nakano, Noriko Motoi, Junichi Tomomatsu, Tabu Gokita, Keisuke Ae, Taisuke Tanizawa, Seiichi Matsumoto, Shunji Takahashi

**Affiliations:** 1 Department of Medical Oncology, Cancer Institute Hospital of the Japanese Foundation for Cancer Research, Ariake, Tokyo, 135-8550 Japan; 2 Division of Pathology, Cancer Institute of the Japanese Foundation for Cancer Research, Tokyo, Japan; 3 Department of Orthopedic Surgery, Cancer Institute Hospital of the Japanese Foundation for Cancer Research, Tokyo, Japan

**Keywords:** Soft tissue sarcoma, Tyrosine kinase inhibitor, Pazopanib, Pneumothorax

## Abstract

**Background:**

After the approval of pazopanib for the treatment of soft tissue sarcoma (STS), pneumothorax was reported as an unexpected adverse event during pazopanib treatment. The incidence and risk factors of pneumothorax during pazopanib treatment for STSs have not been established yet.

**Methods:**

We retrospectively reviewed the cases of all of the STS patients treated with pazopanib between November 2012 and December 2014 at our institute and evaluated the prevalence, incidence, treatment details and risk factors for pneumothorax in the STS patients during pazopanib treatment.

**Results:**

A total of 58 patients were enrolled; 45 of them had lung and/or pleural lesions at the start of pazopanib treatment. During the median follow-up time of 219 days (range 23–659), 13 pneumothorax events occurred in six patients; the prevalence and incidence of pneumothorax were 10.3 % and 0.56 per treatment-year, respectively. The median onset of pneumothorax was day 115 (range 6–311). No patients died of pneumothorax, but pazopanib was interrupted in 10 events and chest drainage was performed in eight events. Pazopanib continuation or restart after the recovery from pneumothorax was conducted after 9 of the 13 events. The median progression-free survival of patients with and without pneumothorax events were 144 and 128 days (*p* = 0.89) and the median overall survival periods were 293 and 285 days (*p* = 0.69), respectively. By logistic regression analyses, the maximum diameter of the lung metastases ≥ 30 mm (OR 13.3, 95 % CI 1.1–155.4, *p* = 0.039) and a history of pneumothorax before the pazopanib induction (OR 16.6, 95 % CI 1.1–256.1, *p* = 0.045) were significantly predictive of pneumothorax.

**Conclusions:**

In our retrospective analysis, pneumothorax was observed in 10.3 % of 58 STS patients during pazopanib treatment. The diameter of the lung metastases and a history of pneumothorax could be useful for evaluating the risk of pneumothorax in pazopanib treatment.

## Background

Soft tissue sarcomas (STSs) are heterogeneous malignant diseases, originating from mesenchymal tissues all over the body. Approximately 30 % of all STS patients have some metastatic lesions, and the prognoses of metastatic STS patients are still poor [1–3]. There have been some case reports of pneumothorax as a complication in STS patients with lung metastases; due to the rarity of the event, however, information about the prevalence and the risk factors of pneumothorax in STS patients has been limited [4].

In 2012, pazopanib, a multitarget tyrosine kinase inhibitor, was approved for the treatment of STS patients based on the evidence obtained in a phase 3 clinical trial, in which pazopanib was shown to improve the prognoses of advanced STS patients [4]. However, throughout the more than 2 years after pazopanib’s approval, pneumothorax has been reported as an unexpected adverse event in STS patients [5, 6]. Though the relation between pneumothorax and pazopanib treatment is not clear, once pneumothorax occurs, in most cases pazopanib treatment would have to be interrupted. For the safe management of pazopanib treatment, it is necessary to evaluate the prevalence, the incidence and the risk factors for pneumothorax in STS patients during pazopanib treatment. Here we investigated the details of pneumothorax events observed in STS patients during pazopanib treatment.

## Methods

This study was approved by the ethics committee of Cancer Institute Hospital of Japanese Foundation for Cancer Research. After the approval of the institutional review board, we retrospectively reviewed the medical records of STS patients treated with pazopanib at our institute between November 2012 and December 2014. We determined the prevalence, the incidence, the severity and the managements of pneumothorax during these patients’ pazopanib treatment. The prevalence of pneumothorax was calculated as the percentage of patients suffering from pneumothorax. The incidence of pneumothorax was calculated as the number of pneumothorax episodes per treatment-year. The severities of pneumothorax events were evaluated by grading based on the U.S. National Center Institute Common Terminology Criteria for Adverse Events (CTCAE version 4.0).

We also reviewed the baseline characteristics of all of the STS patients enrolled in the study and evaluated the clinical risk factors of pneumothorax by comparing the characteristics of the patients with and without pneumothorax events. We performed univariate and the multivariable analyses to evaluate the association between each risk factor and pneumothorax using Fisher’s extract test and a logistic regression test, respectively.

For the evaluation of prognoses, the progression-free survival (PFS) and the overall survival (OS) from the date of pazopanib induction were estimated by the Kaplan-Meier method. The PFS and the OS of the patients with and without pneumothorax events were compared by the log-rank test.

The patients’ objective responses were also evaluated and compared. The objective response and the disease progression were defined based on the Response Evaluation Criteria in Solid Tumors (RECIST) version 1.1. Independent of the objective response, cavitations of lung lesions during pazopanib treatment were also evaluated.

In all analyses, the p-values were two-sided and considered significant when <0.05.

## Results

A total of 58 STS patients had been treated with pazopanib at our institute between November 2012 and December 2014, and the median follow-up time from the start of pazopanib treatment was 219 days (range 23–659 days). At the time of our analyses, 43 patients were certified as showing disease progression and 30 patients had died due to their STS.

The patients’ characteristics at baseline are shown in Table 1. In Japan, pazopanib is also approved for liposarcoma treatment, and we thus included nine liposarcoma patients in the study. Lung and/or pleural lesions were present at baseline in 45 patients (78 %); lung lesions were present in 41 (71 %) patients, and pleural lesions were present in 23 (40 %) patients. Twenty (34 %) patients had smoking histories; details of smoking index (the number of cigarette-years) were as follows; median 238, range (64–1170), and smoking index was 400 or more in 6 patients.

**Table 1:** Characteristics of the 58 STS patients treated with pazopanib

Characteristics	No.	Percentage
Age		
Median	52	
Range	19–72	
≥ 60 year	19	33
Gender		
Male	28	48
Female	30	52
ECOG performance status		
0	35	60
1	23	40
2 or more	0	0
Smoking history		
Absent	38	66
Present	20	34
Smoking index		
Median	238	
Range	64–1170	
≥ 400	6	10
Hypertension		
Absent	48	83
Present	10	17
Pathological diagnoses		
Leiomyosarcoma	13	22
Synovial sarcoma	9	16
Liposarcoma	9	16
Other histologies	27	46
Primary site of disease		
Extremities	21	36
Non-extremities	37	64
Pulmonary disease		
Lung lesions present	41	71
Pleural lesions present	23	40
Number of lung lesions		
1	6	10
2–5	16	28
6–9	6	10
> 10	13	22
Maximum diameter of lung lesions		
< 10 mm	2	3
10–20 mm	11	19
20–30 mm	7	12
30–50 mm	8	14
> 50 mm	13	22

### The prevalence, incidence and management of pneumothorax

Throughout the follow-up period, 13 pneumothorax events were observed in six of the 58 STS patients enrolled in the analysis; the prevalence of pneumothorax was 10.3 %. The median pazopanib treatment period was 115 days (range 5–659 days), and the incidence of pneumothorax was 0.56 per treatment-year. The median onset of pneumothorax events was at 115 days of pazopanib treatment. All patients with pneumothorax events had lung and/or pleural lesions at the start of pazopanib.

**Fig. 1:**
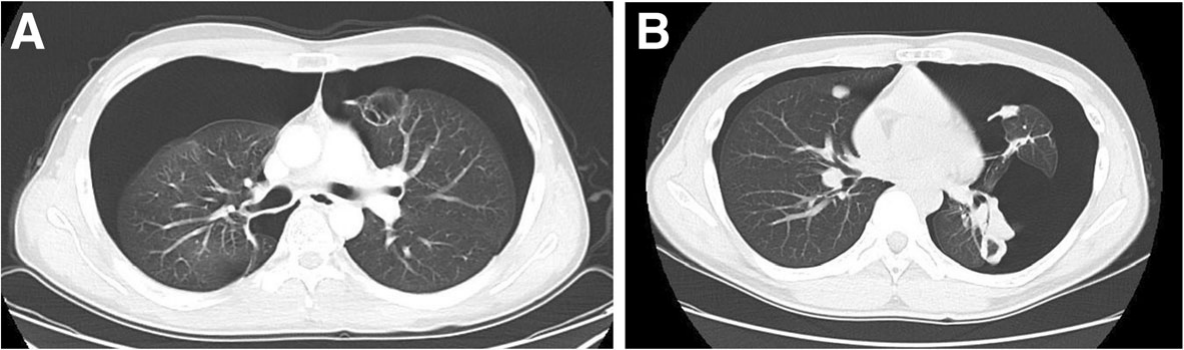
Radiographs of two bilateral pneumothorax events. **a** A bilateral pneumothorax in Patient 3 on treatment day 61. In this patient, cavitations of lung lesions were observed. **b** Bilateral pneumothorax events of Patient 4 on treatment day 95. In the CT scan, progression of lung lesions was also observed

**Fig. 2:**
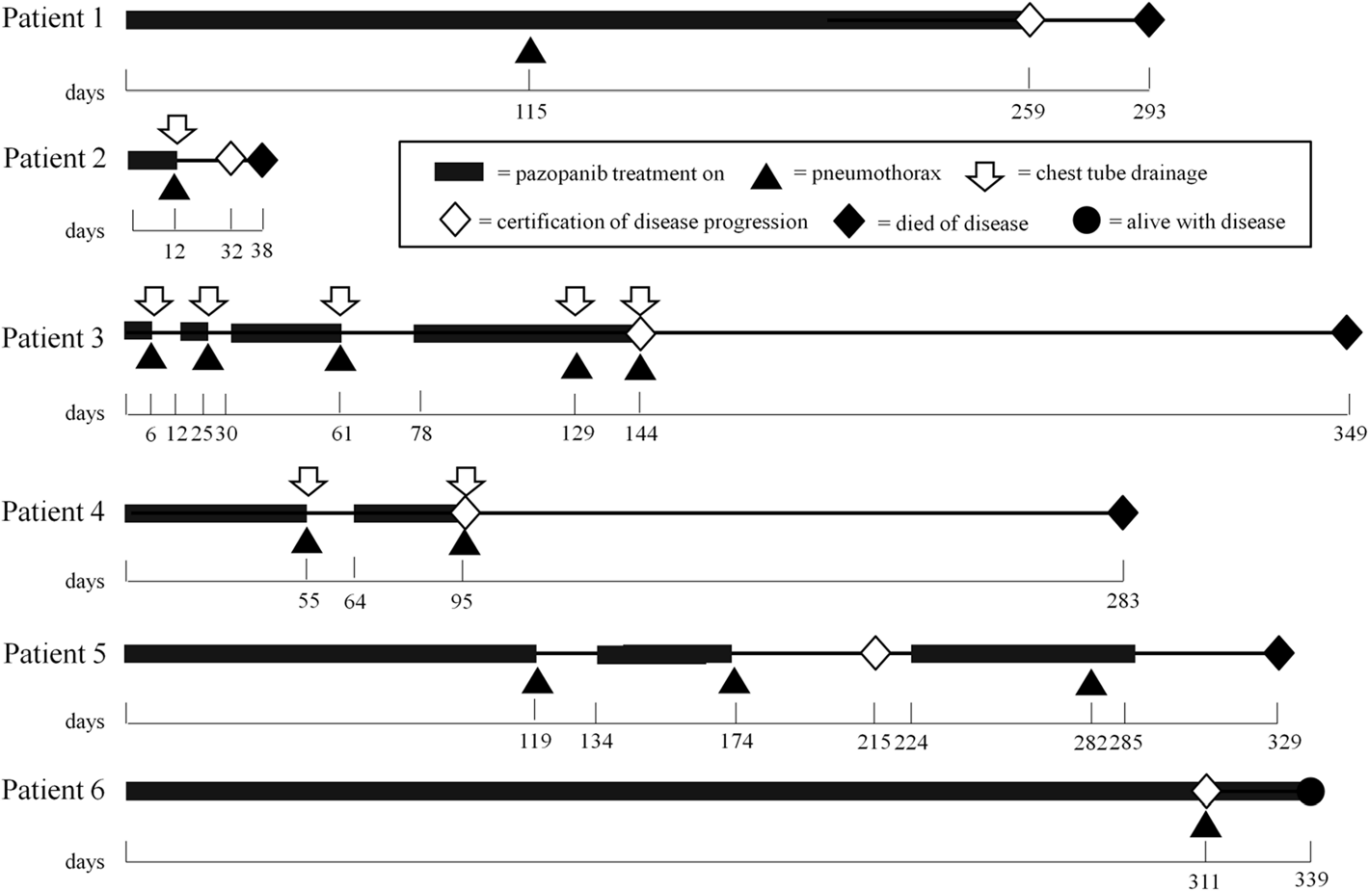
Clinical courses of each of the six patients who had pneumothorax episodes during pazopanib treatment

The details of pneumothorax events are shown in Table 2. Based on the CTCAE, 7 of the 13 pneumothorax events were evaluated as grade 3, and in two events pneumothorax occurred bilaterally (Fig. 1). Recurrences of pneumothorax events were observed in three patients. In Patient 3, a 27-year-old male with undifferentiated sarcoma, not otherwise specified (NOS), pneumothorax events occurred five times during pazopanib treatment. No patients died of pneumothorax, but pazopanib treatment was interrupted in 10 events and the chest drainage was performed in eight events. Pazopanib continuation or restart after the recovery from pneumothorax was conducted after nine events. The clinical courses of the six patients with pneumothorax are summarized in Fig. 2.

**Table 2:** Details of pneumothorax events during pazopanib treatment

Patient characteristics			Clinical status at the occurrence of pneumothorax			
No.	Age	Pathological diagnosis	Treatment day	Grade	Bilateral	Chest drainage
1	60’s	Undifferentiated sarcoma, NOS	115	1	No	No
2	40’s	Carcinosarcoma	12	3	No	Yes
3	20’s	Undifferentiated sarcoma, NOS	6	3	No	Yes
			25	3	No	Yes
			61	3	Yes	Yes
			129	2	No	Yes
			144	3	No	Yes
4	20’s	Synovial sarcoma	55	3	No	Yes
			95	3	Yes	Yes
5	50’s	Synovial sarcoma	145	1	No	No
			174	2	No	No
			282	1	No	No
6	30’s	Synovial sarcoma	311	2	No	No

**Fig. 3:**
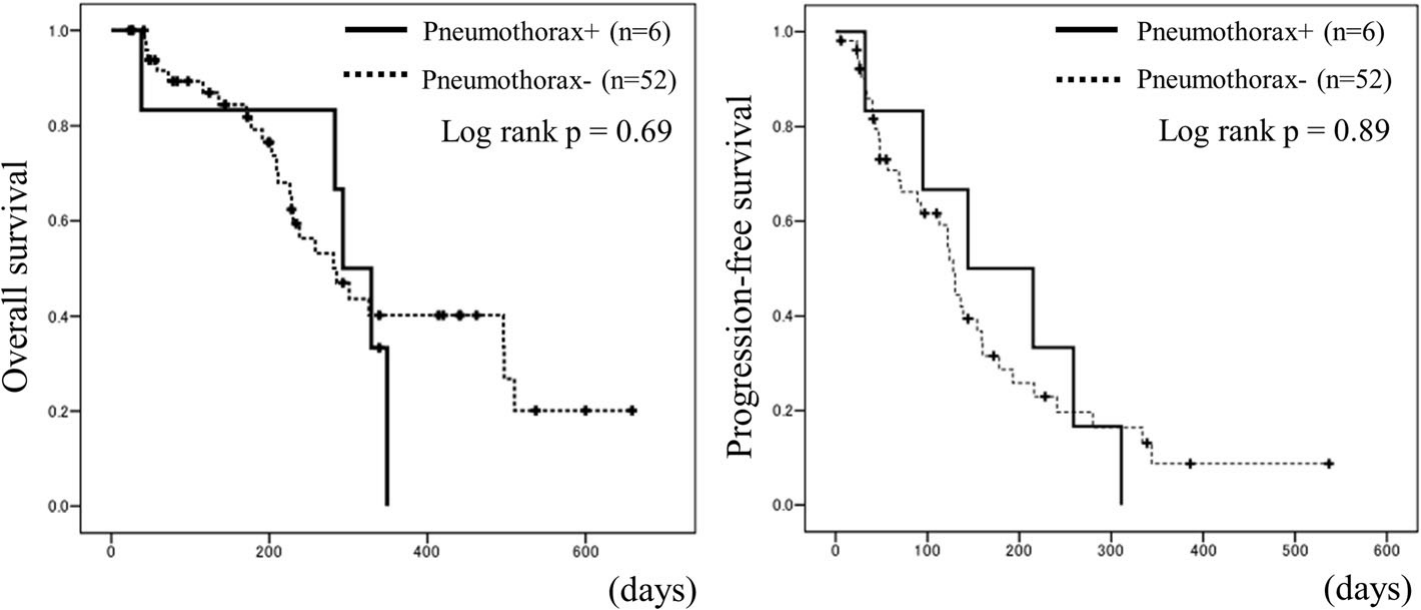
Prognoses of the patients with or without pneumothorax. Prognoses of the patients with or without pneumothorax: overall survival (OS) and progression-free survival (PFS)

### Risk factors of pneumothorax

In our univariate analysis of baseline characteristics of the STS patients treated with pazopanib, the pathological diagnosis of synovial sarcoma, the presence of lung lesions with ≥ 30 mm dia. and the presence of histories of pneumothorax were significant (Table 3). Of these, the multivariable analysis revealed that the maximum diameter of the lung metastases ≥ 30 mm (adjusted odds ratio [OR] = 13.3, 95 % confidence interval [CI] = 1.1–155.4, *p* = 0.039) and the presence of a history of pneumothorax before the pazopanib induction (adjusted OR 16.6, 95 % CI 1.1–256.1, *p* = 0.045) were also significantly predictive of pneumothorax (Table 4).

**Table 3:** Univariate analyses of risk factors of pneumothorax in STS patients during pazopanib treatment

Pneumothorax	Present (*n* = 6)		Absent (*n* = 52)		
Variants	No.	%	No.	%	*p*-value
Gender					
Male	5	83	29	56	0.097
Female	1	17	23	44	
Pathological diagnosis					
Synovial sarcoma	3	50	6	12	0.042
Others	3	50	46	88	
Hypertension					
Present	1	17	9	17	1.00
Absent	5	83	43	83	
Smoking history					
Present	3	50	17	33	0.41
Absent	3	50	35	67	
Smoking index ≥ 400					
Present	2	33	4	8	0.11
Absent	4	67	48	92	
Lung lesion					
Present	6	100	35	67	0.17
Absent	0	0	17	33	
Pleural lesion					
Present	4	67	19	37	0.20
Absent	2	33	33	63	
No. of lung lesions					
0–1	0	0	29	56	0.072
2 or more	6	100	23	44	
Maximum dia. of lung lesions					
≥ 30 mm	5	83	16	31	0.02
< 30 mm	1	17	36	69	
History of lung surgery					
Present	2	33	19	37	1.00
Absent	4	67	33	63	
History of pneumothorax					
Present	2	33	2	4	0.049
Absent	4	67	50	96

**Table 4:** Multivariable analyses of risk factors of pneumothorax in STS patients during pazopanib treatment

Variate	Adjusted Odds ratio	95 % CI	*p*-value
Maximum dia. of lung lesions ≥ 30 mm	13.3	1.1–155.4	0.039
History of pneumothorax	16.6	1.1–256.1	0.045

### Prognoses and responses to pazopanib in STS patients with and without pneumothorax

The median PFS and OS of all STS patients treated with pazopanib were 130 days (95 % CI 112–148) and 285 days (95 % CI 256–313). The comparison of prognoses between the patients with and without pneumothorax by log-rank test showed that the median PFS of the patients with and without pneumothorax were 144 and 128 days (*p* = 0.89) and the median OS values were 293 and 285 days (*p* = 0.69), respectively (Fig. 3). There were no significant differences in PFS or OS due to the presence of pneumothorax events. Prognostic factors other than the presence of pneumothorax were also evaluated by the log-rank test, but there were no statistically significant factors (Table 5).

**Table 5:** The evaluations of prognostic factors other than the presence of pneumothorax by the log-rank test

		Progression-free survival (PFS)		Overall survival (OS)	
Variants	n	Hazard ratio (95 % CI)	*p*-value	Hazard ratio (95 % CI)	*p*-value
Age ≥ 60 yo	19	0.64 (0.33–1.25)	0.19	0.88 (0.40–1.92)	0.74
Male gender	30	1.01 (0.55–1.85)	0.99	1.43 (0.69–2.96)	0.34
Synovial sarcoma	9	0.57 (0.24–1.35)	0.17	0.58 (0.20–1.66)	0.31
Hypertension+	10	0.44 (0.17–1.13)	0.09	1.15 (0.43–3.03)	0.79
Smoking history+	20	0.96 (0.51–1.80)	0.89	1.41 (0.67–2.94)	0.36
lung lesion+	41	1.18 (0.59–2.35)	0.64	2.02 (0.77–5.30)	0.15
Number of lung lesions ≥2	35	0.96 (0.52–1.78)	0.90	1.06 (0.497–2.279)	0.87
Maximum diameter of lung lesions ≥ 30 mm	21	1.37 (0.75–2.51)	0.30	1.40 (0.685–2.878)	0.36

As for the objective responses, a partial response (PR) was observed in 5 of the 58 STS patients (the response rate was 8.6 %); there were no PRs in the patients with pneumothorax (based on the RECIST criteria). Cavitations of lung lesions were observed in four patients, and two of them had pneumothorax during pazopanib treatment (Patient No. 1 and No. 3).

## Discussion

The lung is the major organ in which STS metastases are most often observed; 22 % of all STS patients have lung metastases [7]. Though there have been many case reports of STS patients with pneumothorax, the prevalence and the risk factors of pneumothorax in STS patients have not yet been established.

Hoag et al. reviewed case reports of pneumothorax in STS patients and estimated that the prevalence of pneumothorax in STS patients is 1.9 % [8]. this prevalence is higher than those of patients with primary lung cancers, which was estimated as 0.32 % in Lai’s retrospective analysis [9].

During pazopanib treatment, the prevalence of pneumothorax in STS patients might be higher. In 2014, we preliminarily reported the prevalence of pneumothorax in 32 STS patients treated by pazopanib as 9.4 % [5], and in the present study the prevalence of pneumothorax among 58 STS patients was 10.5 %. Similar to our results, Verschoor et al. reported case series of pneumothorax in STS patients treated by pazopanib; 6 of 43 patients experienced pneumothorax in their study (14.0 %) [6]. In our present study, the prevalence of pneumothorax was even higher than those in prospective clinical trials or multicenter analyses of pazopanib-treated STS patients. In the Palette study, a multicenter phase III trial of pazopanib treatment for STS patients, the prevalence of pneumothorax was 3 % (8 of 246 patients) [4]. In the post-marketing surveillance of pazopanib in Japan, pneumothorax was observed in 23 of 539 patients who received pazopanib (4.3 %), and 12 patients (2.2 %) were diagnosed as grade 3 or more [10].

At our institute, pneumothorax events occurred soon after the approval of pazopanib, and since then chest radiographs have been performed once or twice monthly during pazopanib treatment in clinical practice. This frequent evaluation by chest radiographs helps identify low-grade pneumothorax without clinically relevant symptoms, and it might be the reason for the high prevalence of pneumothorax in our present analysis; in fact, if we exclude the patients with only low-grade pneumothorax (Patient Nos. 1, 5 and 6), the prevalence of severe pneumothorax at our institute was 3 of 58 patients (5.2 %). This result is similar to the prevalence of pneumothorax in the Palette study.

Pazopanib is considered an antiangiogenic agent since it targets the vascular endothelial growth factor receptor (VEGFR) [4]. Antiangiogenic agents are known to cause cavitations of lung lesions [11, 12]. It has been suggested that tumor cavitations during or after chemotherapy might be signs of the clinical response, but also that they could be risk factors for pneumothorax [13]. Our present population included patients in whom cavitations of lung lesions developed during pazopanib treatment, especially among the patients with pneumothorax events. In STS patients, however, cavitations of lung lesions have also occurred during chemotherapies using cytotoxic agents, and it is thought that the necrosis of pulmonary or pleural lesions in response to chemotherapy by cytotoxic agents could be responsible for pneumothorax [8, 14]. Moreover, in the prospective clinical trials of pazopanib treatment for malignancies other than STS, such as renal cell carcinomas and ovarian cancers, pneumothorax was more rarely reported as an adverse event [15, 16]. As for STS patients, the nature of the disease could be more closely related to risk factors of pneumothorax than are the treatment drugs.

In our current analysis, the clinical features of maximum lesion size and number of lung lesions were significant predictors of pneumothorax in the multivariable analysis. However, our analysis was retrospective study, and, due to the small sample size and few numbers of events, the range of adjusted odd ratios were broad (Table 4). These are the limitations of our study, and the re-analyses of bigger sample size, prospective cohorts will be necessary for the certification of risk factors of pneumothorax. In other studies, good performance status and a normal hemoglobin level were suggested to be advantageous for long-term outcomes, and older age was suggested to be associated with liver toxicity [17, 18]. By updating STS patients’ clinical information and analyses, it could be possible to estimate the risk of pneumothorax more precisely in the future.

## Conclusion

Pneumothorax was observed in 10.3 % of 58 STS patients during pazopanib treatment. By the multivariable analyses, the diameter of the lung metastases and a history of pneumothorax could be useful for evaluating the risk of pneumothorax in pazopanib treatment.

## Abbreviations

NOS: Not otherwise specified
OR: Odds ratio
OS: Overall survival
PFS: Progression-free survival
PR: Partial response
STS: Soft tissue sarcoma
VEGFR: Vascular endothelial growth factor receptor
